# Altered Grey Matter Volume and Cerebral Perfusion over the Whole Brain in Painful Temporomandibular Disorders: A Pilot Voxel-Based Analysis

**DOI:** 10.2174/0115734056373583250531004637

**Published:** 2025-06-10

**Authors:** Xin Li, Yujiao Jiang, Zhiye Chen

**Affiliations:** 1 Department of Radiology, Hainan Hospital of PLA General Hospital, Sanya 572015, China; 2 Department of Nuclear Medicine, Xinqiao Hospital, Army Medical University, Chongqing 400037, China; 3 School of Medical Imaging, Bengbu Medical College, Bengbu, Anhui 233030, China

**Keywords:** Temporomandibular disorders, Pain, Brain structure, Perfusion, Voxel-based analysis

## Abstract

**Background:**

Pain with a persistent and recurrent onset is one of the most important symptoms of temporomandibular disorders (TMD). Recent evidence indicated the dysfunction of the central nervous system was more linked to TMD pain. This study aimed to explore the abnormal structural and perfusion alterations in patients with painful TMD (p-TMD) to understand the comprehension of neuro-pathophysiological mechanisms.

**Methods:**

Forty-one p-TMD patients and 33 normal controls (NC) were recruited, and high-resolution structural brain and 3D PCASL data were obtained from a 3.0T MR scanner. The voxel-based analysis of the whole cerebral gray matter (GMV) was performed, and the GMV and cerebral blood flow (CBF) value of the altered positive areas were extracted to investigate the significant correlation with clinical variables.

**Results:**

The brain regions with significantly increased GMV in p-TMD group were listed as follows: right putamen, right superior frontal gyrus, left superior frontal gyrus medial segment, right supplementary motor cortex, left postcentral gyrus, right middle temporal gyrus, right postcentral gyrus medial segment, right temporal pole, right inferior temporal gyrus and right opercular part of the inferior frontal gyrus (*Punc*<0.001, cluster>39). However, there were no brain regions with significantly decreased GMV in the p-TMD group. Cerebral perfusion analysis identified that only the right postcentral gyrus medial segment presented significantly higher CBF value in the p-TMD group than in the NC group over all the brain regions with increased GMV. Within the p-TMD group, pain intensity, anxiety, depression, and jaw functional limitation scores were differentially associated with GMV and CBF value.

**Conclusion:**

The voxel-based morphometric and perfusion findings collectively implicate maladaptive plasticity in both the sensory-discriminative and affective-motivational dimensions of pain processing in p-TMD pathophysiology.

## 1 BACKGROUND

Temporomandibular disorders (TMD) represent a heterogeneous group of conditions affecting the temporo-mandibular joint (TMJ), masticatory musculature, and/or their associated structures [1]. As the overarching diagnostic category for temporomandibular pain and dysfunction, TMD affects approximately 31% of adults and 11% of children [2]. Chronic pain, a hallmark clinical feature of TMD, typically persists for ≥3 months and is exacerbated by TMJ dislocation or palpation [3]. Although painful TMD (p-TMD) constitutes the majority of cases requiring clinical intervention, its underlying pathophysiology remains poorly understood [4]. Consequently, elucidating the mechanistic basis of p-TMD is crucial for developing targeted therapeutic strategies and improving clinical management.

Initially, TMD-related pain was primarily attributed to peripheral mechanisms, wherein repetitive muscle strain or TMJ overloading was believed to trigger increased neuro-peptide release and inflammatory mediator production, ultimately creating a local hypoxic environment that induced pain [5]. However, emerging evidence indicates that chronic pain manifestations are associated with significant neuronal activity changes that lead to subsequent structural and functional brain reorganization [6-8]. Neuroimaging studies have provided substantial insights into these central nervous system alterations in p-TMD patients. According to a structural study, patients with chronic myofascial temporomandibular pain exhibited either increased or decreased gray matter volume (GMV) in various areas of the trigeminothala-mocortical pathway, including brainstem trigeminal sensory nuclei, when compared to normal controls (NC) [9]. These findings suggest that brain structural assessment could serve as a valuable complementary approach for evaluating neuronal dysfunction in p-TMD pathophysiology.

Structural brain changes frequently co-occur with functional alterations. Existing research demonstrates that sustained nociceptive input can induce changes in central pain processing pathways. These neuroplastic adaptations may be reflected in regional blood perfusion variations, potentially attributable to neuronal activity or density modifications [10]. Given the well-established neurovascular coupling mechanism linking neural activity to cerebral perfusion and metabolism, these changes are particularly relevant. Several functional MRI studies revealed significant disruptions in corticostriatal circuitry in TMD patients, along with abnormal pain-related spontaneous activity and functional connectivity patterns in temporomandibular joint synovitis pain cases [11, 12]. To quantitatively assess these perfusion changes, we will employ 3D pseudo-continuous arterial spin labeling (3D PCASL), an advanced perfusion imaging technique that enables non-invasive quantification of cerebral blood flow (CBF) without contrast administration [13]. This multimodal neuroimaging approach will allow a comprehensive investigation of p-TMD-related neurobiological changes across structural, functional, and hemodynamic domains.

To investigate potential neurobiological alterations in p-TMD, we hypothesized that patients would exhibit distinct patterns of brain structural and perfusion abnormalities. To test this hypothesis, we sequentially recruited 41 p-TMD patients and 33 age- and sex-matched normal controls (NCs). The voxel-based analysis (VBA) was employed to comprehensively assess GMV and CBF changes, providing insights into the underlying pathophysiology of p-TMD. Furthermore, correlation analyses were conducted to explore associations between clinical symptom severity and neuroimaging-derived metrics.

## 2 METHODS

### 2.1 Subjects

This study was conducted in accordance with the ethical principles outlined in the Declaration of Helsinki and was approved by the Institutional Review Board of Hainan Hospital, PLA General Hospital (Ethics Approval No. S2022-03). Written informed consent was obtained from all participants prior to their inclusion in the study.

Inclusion criteria for the p-TMD group were listed as follows: (1) All individuals were examined and diagnosed with TMD by one senior specialist using the Diagnostic Criteria for Temporomandibular Disorders (DC/TMD), which is the most widely accepted and standardized tool for assessment and classification of TMD [14, 15]; (2) Pain in the TMJ, masticatory musculature, or head caused by TMD during rest or function; (3) Being in the interictal stage at least three days after a pain attack for TMD; (4) Never receiving any treatment in the past two weeks, such as psychotherapy, physiotherapy, joint injection; (5) Avoiding the alcohol, nicotine, caffeine and other substances at least 12 hours before MRI examinations; (6) At least 18 years of age and right-handed. And the age- and gender-matched NC should never have had any primary TMJ-related diseases or other types of pain in the past years. The exclusion criteria for all participants should include the following: neurological disorder, vascular disease, neoplasia, pregnancy, metabolic disease, psychiatric disorders, and MRI contraindications, including metal clips within the body and claustrophobia.

All participants underwent comprehensive clinical assessments using Axis II of the DC/TMD. The evaluation included: Jaw Functional Limitation Scale (JFLS), Patient Health Questionnaire-9 (PHQ-9) for depressive evaluation, Generalized Anxiety Disorder-7 (GAD-7) for anxiety evaluation, Patient Health Questionnaire-15 (PHQ-15) for somatization symptoms evaluation, and Oral Behavior Checklist (OBC) for evaluation of oral bad behavior frequency. Additionally, standardized clinician-administered scales were employed: Hamilton Anxiety Scale (HAMA) and Hamilton Depression Scale (HAMD). Pain intensity was specifically assessed in p-TMD patients using the Visual Analog Scale (VAS).

### 2.2 MRI Data Acquisition

MRI data were collected using a Philips 3.0T MR system (Ingenia CX, Netherlands) with a thirty-two-channel brain receiving coil. MR sequences included as follows: (1) a three-dimensional T1-weighted fast field echo (3D T1WI-FFE) sequence: TR/TE = 6.6/3.0ms, matrix = 220 × 240, field of view (FOV) = 22 cm× 24 cm, 150 axial slices with 1mm thickness, yielding a voxel size of 1 × 1 × 1 mm; (2) the cerebral perfusion imaging by a 3D pseudo-continuous arterial spin labeling (3D PCASL) with the following setting: TR/TE = 4252/11ms, flip angle = 90°, matrix = 64 × 60; FOV=24 cm×24 cm, slice thickness = 5.0 mm, and post-labeling delay time (PLD) = 2000ms. The 27 slices of CBF images could be automatically generated by using Functional tools (version: 9.4.05) after PCASL scanning [16].

### 2.3 Data Processing

All the imaging data were processed with voxel-based analysis using Statistical Parametric Mapping 12 (SPM12) (Wellcome Department of Cognitive Neurology, London, UK) (https://www.fil.ion.ucl.ac.uk/spm/), which was run under MATLAB 2021b (The Mathworks, Natick, MA, USA).

**Fig. (1):**
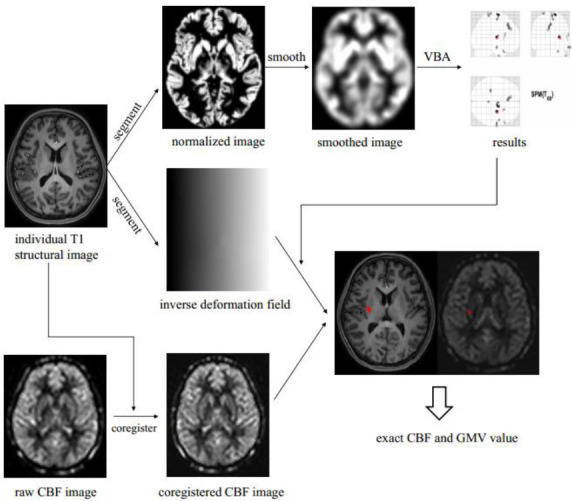
Preprocessing steps for voxel-based analysis and the CBF, GMV value extraction.

Image processing included the following steps (Fig. **1**): (1) Set origin: all the raw image origin was set at anterior commissure (0, 0, 0); (2) Segmentation: the individual T1 structural images were segmented by the new segment tool embedded in SPM 12 software and the inverse deformation field (IDF) was generated; (3) Data quality control and get total intracranial volume (TIV): The quality control of the data was performed, and four p-TMD patients had been excluded. (4) Normalization: The individual segmented images were normalized to a standard template (Montreal Neurological Institute); (5) Smoothening: 8mm full width at half maximum was applied to normalized images with Gaussian smoothing spatially; (6) Voxel-based analysis; (7) Generate the individual regions: the standard positive regions were applied with the IDF with pullback strategy, which would generate the individual positive regions; (8) Coregistered CBF images: the raw CBF images were coregistered with the raw 3D T1WI (3D T1-FFE), and then generated coregistered CBF images; (9) GMV and CBF value calculation: calculate the individual positive regions volume and perfusion using ITK-SNAP software (https://earth.esa.int/eogateway/tools/snap).

### 2.4 Statistical Analysis

An independent two-sample t-test was used to investigate the positive regions with significant volume differences in p-TMD patients compared with the NC, with age, gender and TIV as covariates. Statistical significance was set at an uncorrected voxel-level threshold of p < 0.001, with cluster-level inference based on expected spatial extent [17, 18]. Only clusters surviving these combined thresholds were retained for further analysis, while nonsignificant clusters were excluded.

The clinical data was processed by IBM-SPSS 25. The quantitative data with normal distribution are presented as mean ± SD, and the quantitative data with non-normal distribution are presented by median (the first quartile, the third quartile). The quantitative variables were analyzed with an independent sample *t* test, and the qualitative variable was analyzed with the Chi-Square test. The correlation analysis was performed with the Pearson method for the data with normal distribution and with the Spearman method for non-normal distribution. The *Pvalue* of less than 0.05 was considered to indicate a statistically significant difference.

## 3 RESULT

### 3.1 Demography and Clinical Characteristics

This study enrolled 41 p-TMD patients and 33 NCs, and demographic and clinical characteristics were presented in Table **1**. No significant between-group differences were observed in age, sex distribution, or psychological measures including HAMA, HAMD, GAD-7, PHQ-9, PHQ-15, and OBC scores (all p>0.05). However, p-TMD patients had a significantly higher JFLS score (35.00(50.00, 16.00)) than NC (3.00(6.50, 0.00)) (*P*<0.05). The p-TMD group reported moderate pain levels, with a median VAS score of 3.00 (4.50-2.00).

### 3.2 The Brain Regions with Increased GMV in the p-TMD Group

**Fig. (2):**
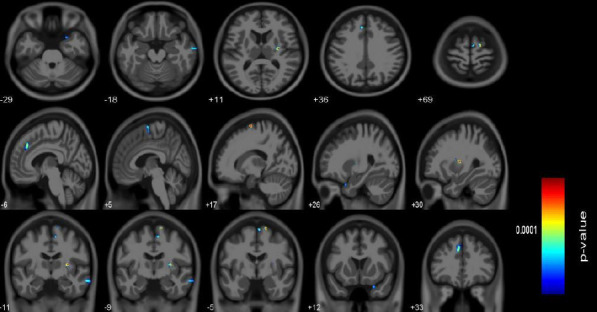
The brain regions with increased gray matter volume over the whole brain in TMD pain group.

The p-TMD patients presented increased GMV, and the involved positive brain regions mainly included as follows (Table **2** and Fig. **2**): right putamen, right superior frontal gyrus (Right SFG), left superior frontal gyrus medial segment (Left MSFG), right supplementary motor cortex (Right SMC), left postcentral gyrus (Left PoG), right middle temporal gyrus (Right MTG), right postcentral gyrus medial segment (Right MPoG), right temporal pole (Right TMP), right inferior temporal gyrus (Right ITG), right opercular part of the inferior frontal gyrus (Right OpIFG)(*P*_unc_<0.001, cluster>39). p-TMD did not present significantly decreased GMV compared to NC.

### 3.3 The Brain Regions with Increased CBF in the p-TMD Group

Perfusion analysis was performed for the positive brain regions with increased GMV regions. Table **3** showed that only Right MPoG presented significantly higher CBF value in the p-TMD group than that of in NC group (Table **3**). There were no statistically significant differences in the other regions with increased GMV.

### 3.4 The Correlation Analysis between GMV and CBF Value in the Positive Brain Regions with Increased GMV and the Clinical Characteristics in the p-TMD Group

**Fig. (3):**
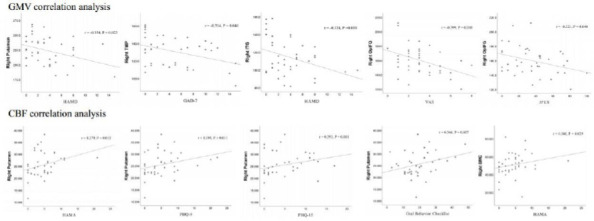
Correlation analysis between the GMV, CBF value with clinical variables of all the brain regions with significant volume difference in TMD pain group.

The GMV correlation analysis demonstrated that HAMD was negatively correlated with the GMV value of right putamen (r = −0.354, *P* = 0.023) and right inferior temporal gyrus (r = −0.324, *P* = 0.039); GAD-7 was negatively correlated with the GMV value of right temporal pole (r = −0.314, *P* = 0.046); VAS and JFLS value were negatively correlated with the GMV value of right opercular part of the inferior frontal gyrus (VAS: r = −0.399, *P* = 0.010; JFLS: r = −0.321, *P* = 0.040) (Table **4** and Fig. **3**).

The CBF correlation analysis revealed that HAMA was positively correlated with the CBF value of right putamen (r = 0.379, *P* = 0.015) and supplementary motor cortex (r = 0.341, *P* = 0.029); PHQ-9, PHQ-15 and OBC were also positively correlated with the CBF value of right putamen (PHQ-9: r = 0.395, *P* = 0.011; PHQ-15: r = 0.393, *P* = 0.011; OBC: r = 0.346, *P* = 0.027) (Table **5** and Fig. **3**).

## 4 DISCUSSION

Advances in computational neuroimaging have enabled voxel-based analysis (VBA) to objectively and precisely quantify regional structural and perfusion abnormalities in the brain. In this study, we identified distinct neurobiological alterations in p-TMD patients compared to normal controls (NCs), characterized by: (1) Widespread GMV increases across multiple brain regions, and (2) Focal CBF hyperperfusion in a single brain region. These neuroimaging findings provide novel insights into the potential neurological mechanisms underlying p-TMD pathophysiology, offering testable hypotheses for future research.

Our study identified neurostructural abnormalities in p-TMD patients, primarily localized to the right putamen, temporal lobe, and bilateral frontal regions. These areas are integral to both sensory-discriminative and affective-motivational dimensions of pain processing. The observed widespread structural alterations may reflect adaptive neuroplastic changes associated with the complex, centralized pain regulation mechanisms in p-TMD.

The putamen, a key structure within the basal ganglia circuitry, plays a significant role in pain discrimination, modulation, and integration of nociceptive signals from ascending pain pathways [19]. Supporting this functional role, neuroimaging studies have demonstrated structural adaptations in the putamen across various chronic pain conditions. Specifically, patients with myofascial TMD show increased GMV in the posterior putamen compared to NCs, suggesting this region's capacity for neuroplastic responses to both nociceptive and non-nociceptive stimuli [9]. Similar putaminal GMV increases have been documented in chronic back pain [20] and chronic vulvar pain [21]. These convergent findings across distinct pain conditions, including our observations in p-TMD, may reflect that the changes in brain volume observed in p-TMD patients may represent somatotopic reorganization associated with persistent pain.

Our study identified increased GMV in the right temporal lobe of p-TMD patients, particularly within the middle and inferior temporal gyri. This finding contrasts with reports of reduced GMV in the left superior/middle temporal gyrus among classic trigeminal neuralgia (CTN) patients [22], where the degree of atrophy negatively correlated with pain intensity and duration [23]. However, in this study, an increase in GMV was found in the temporal lobe of p-TMD patients, which could be due to the different duration of pain. These differential temporal lobe alterations across pain conditions may reflect: (1) disease-stage dependent neuroplasticity, where acute pain may initially induce tissue contraction while chronic pain leads to subsequent reorganization or atrophy [24], and (2) the temporal lobe's dual role in both pain processing and affective regulation through limbic connections. On the other hand, different regions of the temporal lobe could be involved in the generation, control, and regulation of emotions related to the limbic system [25]. Liotti *et al*. found that patients with affective disorders in which anxiety is prevalent are associated with specific activations of the anterior temporal cortex [26]. This is also consistent with the findings of the present study. The abnormal structural alteration of the middle and inferior temporal gyrus in this study may have some relation to the emotional symptoms in p-TMD patients. GMV values of the right temporal pole and inferior temporal gyrus are negatively correlated with anxiety and depression scores. This pattern suggests that temporal lobe structural changes in p-TMD may represent neuroadaptive responses to both persistent nociception and emotional distress.

The frontal lobe is responsible for pain processing by regulating the nociceptive pathways in the cortex and subcortex [27]. Previous neuroimaging studies of chronic pain conditions have demonstrated frontal lobe alterations, including: (1) reduced GMV in the bilateral middle frontal gyrus in episodic cluster headache [28], and (2) decreased ReHo in the superior/middle frontal gyrus in tension-type headache [29]. In contrast, our study found increased GMV in the superior frontal gyrus of p-TMD patients, which may reflect adaptive cortical reorganization in response to persistent nociceptive input. Notably, we observed no significant structural changes in the prefrontal cortex (PFC) - a key region for affective processing [30]. This absence of PFC alterations may correspond to the comparable levels of negative affect (*e.g*., anxiety, depression) between p-TMD patients and NCs. However, future large-scale studies are needed to clarify the differential involvement of frontal subregions in p-TMD pathophysiology and determine whether structural changes represent compensatory mechanisms or maladaptive plasticity.

Cerebral blood flow is intrinsically coupled with neuronal metabolism under physiological conditions, where functionally active brain regions typically exhibit higher perfusion rates reflecting increased metabolic demands and energy consumption [31]. Our study revealed a dissociation between structural and hemodynamic changes in p-TMD patients: while multiple brain regions showed GMV increases, only a single region demonstrated CBF elevation. This spatial discrepancy may reflect distinct temporal phases of neurovascular adaptation. Regional hyperperfusion from increased neuronal activity may precede structural reorganization in the acute phase, while in the chronic phase, sustained pain may trigger homeostatic mechanisms to restore normal perfusion patterns [32]. Previous studies had shown that nociceptive stimuli could result in disturbances in cerebrovascular regulation or differences in the metabolism or activity of neurons [33, 34], and the current study further supported that the abnormal CBF state might be a consequence of the severity and magnitude of the migraine attack. However, the dynamic trajectory of perfusion changes during treatment response remains unknown and warrants longitudinal investigation to determine whether CBF normalization predicts clinical improvement.

Our analyses identified distinct neuroimaging correlations of clinical symptoms in p-TMD patients, revealing differential associations with GMV and CBF. Firstly, the correlation analysis revealed that the VAS score was negatively correlated only with the GMV value of the right opercular part of the inferior frontal gyrus, but there was no significant correlation with the CBF value. Based on this point, it could suggest that the volume change was particularly pronounced in p-TMD patients. In addition, the variables anxiety and depression were negatively correlated with the GMV value in the right putamen, but positively correlated with the CBF value, suggesting that the putamen played a role in the regulation of negative emotions. This could be due to the fact that the basal ganglion had a close functional relationship with the limbic system, which was involved in the integration of emotional and physical states [35]. Finally, the JFLS score was negatively correlated with the GMV value of the right opercular part of the inferior frontal gyrus, suggesting that this region may be the early motor injury brain area involved in TMJ movement regulation.

Limitations of this study include as follows: (1) The moderate sample size may limit the generalizability of our results. Future large-scale studies are needed to validate these neuroimaging findings. (2) This study was a cross-sectional observation, and longitudinal investigations are required to determine whether the structural/functional alterations represent reversible neuroplasticity. (3) The current diagnostic categorization did not differentiate between p-TMD subtypes. Future studies should employ stratified analyses to distinguish neural mechanisms across pain phenotypes and explore potential subtype-specific biomarkers.

## CONCLUSION

In conclusion, while demonstrating widespread GMV alterations, the current study revealed increased CBF specifically localized to a single brain region in p-TMD patients. The focal hyperperfusion pattern, contrasting with distributed structural changes, may represent a compensatory neurovascular response to chronic nociceptive input. These voxel-based morphometric and perfusion findings collectively implicate maladaptive plasticity in both the sensory-discriminative and affective-motivational dimensions of pain processing in p-TMD pathophysiology.

## Figures and Tables

**Table 1:** Demographic and clinical characteristics of the subjects.

	**TMD**	**NC**	**P-value**
Num(M/F)	41(18/23)	33(12/21)	0.511
Age^a^	25.00(33.00, 22.00)	25.00(26.50, 23.00)	0.785
HAMA^a^	4.00(6.00, 2.00)	3.00(6.50, 1.00)	0.450
HAMD^a^	3.00(5.00, 1.00)	2.00(4.00, 1.00)	0.305
GAD-7^a^	3.00(7.50, 0.00)	1.00(5.00, 0.00)	0.157
PHQ-9^a^	4.00(8.50, 1.00)	4.00(7.00, 2.00)	0.710
PHQ-15^a^	3.00(8.50, 0.50)	3.00(5.00, 0.00)	0.353
JFLS^a^	35.00(50.00, 16.00)	3.00(6.50, 0.00)	0.000
OBC^a^	18.00(24.50, 13.50)	15.00(26.00, 11.50)	0.439
VAS^a^	3.00(4.50, 2.00)	NA	NA

**Abbreviations:** HAMA: Hamilton Anxiety Scale; HAMD: Hamilton Depression Scale; GAD-7: Generalized Anxiety Disorder-7; PHQ-9: Patient Health Questionnaire-9; PHQ-15: Patient Health Questionnaire-15; JFLS: Jaw Functional Limitation Scale; OBC: Oral Behavior Checklist; VAS: Visual Analogue Score; NA: Not Available; ^a^Median (the first quartile, the third quartile).

**Table 2:** The brain regions with increased GMV in p-TMD compared with NC.

**Anatomic Region**	**MNI-space**			**Cluster Size**	***P*_uncorr_**	**Peak T-value**
	**X**	**Y**	**Z**			
Right Putamen	30	-11	11	92	0.000	4.47
Right SFG	14	-5	71	94	0.000	4.43
Left MSFG	-6	33	36	300	0.000	3.76
Right SMC	5	-5	69	98	0.000	3.72
Left PoG	-27	-33	75	56	0.000	3.64
Right MTG	63	-11	-18	89	0.000	3.64
Right MPoG	3	-39	69	70	0.000	3.54
Right TMP	26	12	-29	50	0.000	3.51
Right ITG	53	-5	-41	42	0.000	3.41
Right OpIFG	63	18	17	62	0.000	3.33

**Abbreviations:** Right SFG: right superior frontal gyrus; Left MSFG: left superior frontal gyrus medial segment; Right SMC: right supplementary motor cortex; Left PoG: left postcentral gyrus; Right MTG: right middle temporal gyrus; Right MPoG: right postcentral gyrus medial segment; Right TMP: right temporal pole; Right ITG: right inferior temporal gyrus; Right OpIFG: right opercular part of the inferior frontal gyrus.

**Table 3:** The perfusion alternations in the brain regions with significant volume difference in p-TMD and NC group.

**Anatomic regions**	**TMD^b^**	**NC^b^**	**P-value**
Right Putamen	25.486±5.062	23.108±5.275	0.053
Right SFG	47.445±13.277	41.937±10.534	0.056
Left MSFG	55.228±11.057	51.050±11.771	0.121
Right SMC^a^	50.206(44.896, 57.521)	48.676(44.752,53.797)	0.539
Left PoG	44.925±10.899	42.077±14.157	0.331
Right MTG	47.397±10.055	43.815±10.398	0.138
Right MPoG	50.494±10.835	44.844±12.277	0.039
Right TMP	38.284±9.701	35.553±11.995	0.282
Right ITG	42.314±9.139	40.955±11.126	0.565
Right OpIFG	51.737±13.324	49.345±11.031	0.411

**Notes:** ^a^Median (the first quartile, the third quartile); ^b^ ml/100 mg·min. **Abbreviations:** Right SFG: right superior frontal gyrus; Left MSFG: left superior frontal gyrus medial segment; Right SMC: right supplementary motor cortex; Left PoG: left postcentral gyrus; Right MTG: right middle temporal gyrus; Right MPoG: right postcentral gyrus medial segment; Right TMP: right temporal pole; Right ITG: right inferior temporal gyrus; Right OpIFG: right opercular part of the inferior frontal gyrus.

**Table 4:** Correlation analysis between clinical variables and the GMV value of brain regions with significant volume difference.

**Anatomic regions**		**Right Putamen**	**Right** **SFG**	**Left MSFG**	**Right SMC**	**Left** **PoG**	**Right MTG**	**Right MPoG**	**Right TMP**	**Right** **ITG**	**Right OpIFG**
VAS	r	−0.151	−0.083	−0.023	−0.221	−0.107	−0.068	0.012	−0.069	−0.205	−0.399
	*P*	0.347	0.606	0.886	0.165	0.505	0.672	0.942	0.666	0.198	0.010
HAMA	r	−0.115	−0.111	−0.163	−0.094	−0.057	−0.274	0.059	−0.019	−0.222	−0.046
	*P*	0.475	0.489	0.308	0.557	0.725	0.083	0.715	0.908	0.164	0.775
HAMD	r	−0.354	−0.214	−0.184	−0.261	−0.079	−0.300	−0.019	−0.232	−0.324	−0.300
	*P*	0.023	0.180	0.249	0.099	0.621	0.056	0.906	0.144	0.039	0.056
JFLS	r	−0.124	−0.014	−0.085	0.003	−0.064	−0.275	0.014	−0.175	−0.179	−0.321
	*P*	0.439	0.929	0.597	0.984	0.689	0.082	0.928	0.274	0.262	0.040
PHQ-9	r	−0.257	−0.211	−0.136	−0.163	−0.058	−0.203	0.161	−0.184	−0.030	−0.180
	*P*	0.105	0.185	0.396	0.310	0.720	0.203	0.314	0.249	0.853	0.260
GAD-7	r	−0.28	−0.273	−0.206	−0.262	−0.087	−0.222	0.010	−0.314	−0.221	−0.216
	*P*	0.076	0.084	0.195	0.098	0.590	0.163	0.952	0.046	0.165	0.175
PHQ-15	r	−0.257	−0.173	−0.066	−0.108	0.005	−0.175	0.128	−0.109	0.002	−0.100
	*P*	0.105	0.279	0.680	0.501	0.975	0.273	0.426	0.498	0.992	0.535
OBC	r	−0.244	−0.173	−0.129	−0.196	−0.188	−0.285	−0.055	−0.130	−0.090	−0.200
	*P*	0.124	0.280	0.420	0.220	0.240	0.071	0.731	0.417	0.577	0.210

**Abbreviations:** Right SFG: right superior frontal gyrus; Left MSFG: left superior frontal gyrus medial segment; Right SMC: right supplementary motor cortex; Left PoG: left postcentral gyrus; Right MTG: right middle temporal gyrus; Right MPoG: right postcentral gyrus medial segment; Right TMP: right temporal pole; Right ITG: right inferior temporal gyrus; Right OpIFG: right opercular part of the inferior frontal gyrus; VAS: Visual Analogue Score; HAMA: Hamilton Anxiety Scale; HAMD: Hamilton Depression Scale; JFLS: Jaw Functional Limitation Scale; PHQ-9: Patient Health Questionnaire-9; GAD-7: Generalized Anxiety Disorder-7; PHQ-15: Patient Health Questionnaire-15; OBC: Oral Behavior Checklist.

**Table 5:** Correlation analysis between clinical variables and the CBF value of brain regions with significant volume difference.

**Anatomic regions**		**Right Putamen**	**Right** **SFG**	**Left MSFG**	**Right SMC**	**Left** **PoG**	**Right MTG**	**Right MPoG**	**Right TMP**	**Right** **ITG**	**Right OpIFG**
VAS	r	−0.077	−0.027	0.199	0.051	0.114	0.242	0.121	0.049	0.129	−0.061
	*P*	0.632	0.869	0.211	0.751	0.477	0.127	0.451	0.760	0.421	0.703
HAMA	r	0.379	0.288	0.283	0.341	0.112	0.109	0.266	0.086	0.109	0.284
	*P*	0.015	0.067	0.073	0.029	0.485	0.499	0.092	0.592	0.497	0.072
HAMD	r	0.282	0.158	0.196	0.146	−0.048	0.066	0.211	−0.071	−0.017	0.051
	*P*	0.074	0.325	0.220	0.361	0.765	0.683	0.185	0.659	0.915	0.750
JFLS	r	−0.031	0.145	0.151	0.222	0.036	0.104	0.197	0.214	0.143	0.099
	*P*	0.848	0.366	0.347	0.162	0.826	0.517	0.217	0.179	0.371	0.539
PHQ-9	r	0.395	0.042	0.172	0.222	−0.022	0.118	0.239	0.099	0.044	0.085
	*P*	0.011	0.795	0.283	0.162	0.893	0.462	0.132	0.537	0.786	0.597
GAD-7	r	0.290	0.081	0.188	0.155	−0.050	−0.019	0.259	0.050	−0.026	0.047
	*P*	0.066	0.613	0.240	0.332	0.756	0.908	0.102	0.757	0.871	0.771
PHQ-15	r	0.393	0.118	0.204	0.162	0.028	0.066	0.288	−0.025	0.047	0.153
	*P*	0.011	0.464	0.200	0.311	0.860	0.681	0.068	0.877	0.773	0.340
OBC	r	0.346	0.067	0.151	0.193	0.026	0.137	0.193	0.218	0.031	0.233
	*P*	0.027	0.678	0.346	0.225	0.870	0.395	0.228	0.172	0.846	0.142

**Abbreviations:** Right SFG: right superior frontal gyrus; Left MSFG: left superior frontal gyrus medial segment; Right SMC: right supplementary motor cortex; Left PoG: left postcentral gyrus; Right MTG: right middle temporal gyrus; Right MPoG: right postcentral gyrus medial segment; Right TMP: right temporal pole; Right ITG: right inferior temporal gyrus; Right OpIFG: right opercular part of the inferior frontal gyrus; VAS: Visual Analogue Score; HAMA: Hamilton Anxiety Scale; HAMD: Hamilton Depression Scale; JFLS: Jaw Functional Limitation Scale; PHQ-9: Patient Health Questionnaire-9; GAD-7: Generalized Anxiety Disorder-7; PHQ-15: Patient Health Questionnaire-15; OBC: Oral Behavior Checklist.

## Data Availability

The data and supportive information are available within the article.

## LIST OF ABBREVIATIONS

NC: Normal Controls
CBF: Cerebral Blood Flow
VBA: Voxel-based Analysis
VAS: Visual Analog Scale

## STANDARDS OF REPORTING

STROBE guidelines were followed.
